# Anxiety and Depression in Patients with Graves Disease: A Systematic Review

**DOI:** 10.1192/bjo.2026.11119

**Published:** 2026-06-30

**Authors:** Sabah Rehman, Ruihua Hou

**Affiliations:** University of Southampton, Southampton, United Kingdom

## Abstract

**Aims:**

Graves disease (GD) is an autoimmune thyroid disease and is the most common cause of hyperthyroidism in developed countries. Research suggests that patients with GD experience high levels of anxiety and depression. Until recently, this increased prevalence hasbeen attributed largely to Graves-induced hyperthyroidism, however even after achieving euthyroidism, many patients continue to suffer from neuropsychiatric symptoms. This suggests that the mood symptoms present in these individuals may be associated with the autoimmune inflammatory processes underlying GD. This relationship has yet to be fully understood, highlighting the need for an evaluation of the current literature. This systematic review aimed to assess the prevalence of anxiety and depression in GD patients and to identify associated risk factors.

**Methods:**

The review adhered to Preferred Reporting Items for Systematic Reviews and Meta-Analyses (PRISMA) guidelines and was registered on the International Prospective Register of Systematic Reviews (PROSPERO) (CRD42024611942). EMBASE, MEDLINE, PsycINFO and the Cochrane Library were searched up to 31 October 2024. Papers were included providing the participants were diagnosed with GD and satisfied the diagnostic criteria for anxiety, depression or both; or provided symptomatology measures. Studies were quality assessed using Hawker’s tool. 29 studies were included and underwent data extraction. Studies using a case-control design were summarised separately.

**Results:**

Symptoms of anxiety and depression were consistently higher in the GD group versus healthy controls. GD patients displayed a higher baseline degree of anxiety and depression, compared with patients with other causes of hyperthyroidism. Depression and anxiety were more prevalent in relapsed hyperthyroid GD patients, compared with remitted euthyroid GD patients. Risk factors included body mass index (BMI), physical activity, gender, stress and smoking. A limitation of the review was the high study heterogeneity resulting from the variety of study designs included.

**Conclusion:**

These findings highlight a population that may benefit from early psychiatric assessment. The higher prevalence of anxiety and depression observed in GD patients, including those who are euthyroid, reflects the complex interplay between autoimmune, endocrine and psychological processes underpinning the relationship between GD and mental health conditions. This pattern emphasises the need for a multidisciplinary, integrated and holistic approach when managing and treating GD patients. Future research establishing the mechanisms responsible for this increased prevalence could facilitate the development of targeted interventions and preventative treatments.

Funding: None.

